# Mitochondrial Damage and Necroptosis in Aging Cochlea

**DOI:** 10.3390/ijms21072505

**Published:** 2020-04-03

**Authors:** Ah-Ra Lyu, Tae Hwan Kim, Sung Jae Park, Sun-Ae Shin, Seong-Hun Jeong, Yang Yu, Yang Hoon Huh, A Reum Je, Min Jung Park, Yong-Ho Park

**Affiliations:** 1 Department of Otolaryngology-Head and Neck Surgery, College of Medicine, Chungnam National University, Daejeon 35015, Korea; ahmilove@naver.com (A.-R.L.); naong1130@naver.com (S.J.P.); ttd0707@naver.com (S.-A.S.);; 2 Department of Medical Science, College of Medicine, Chungnam National University, Daejeon 35015, Korea; hunpass2@gmail.com; 3 Biomedical Convergence Research Center, Chungnam National University Hospital, Daejeon 35015, Korea; czkth@naver.com; 4 Brain Research Institute, College of Medicine, Chungnam National University, Daejeon 35015, Korea; 5 Electron Microscopy Research Center, Korea Basic Science Institute, Cheongju 28119, Korea; hyh1127@kbsi.re.kr (Y.H.H.); areum83@kbsi.re.kr (A.R.J.)

**Keywords:** hearing loss, age-related, regional blood flow, mitochondria, necroptosis, cochlea

## Abstract

Age-related hearing loss (ARHL) is an irreversible, progressive neurodegenerative disorder and is presently untreatable. Previous studies using animal models have suggested mitochondrial damage and programmed cell death to be involved with ARHL. Thus, we further investigated the pathophysiologic role of mitochondria and necroptosis in aged C57BL/6J male mice. Aged mice (20 months old) exhibited a significant loss of hearing, number of hair cells, neuronal fibers, and synaptic ribbons compared to young mice. Ultrastructural analysis of aged cochleae revealed damaged mitochondria with absent or disorganized cristae. Aged mice also showed significant decrease in cochlear blood flow, and exhibited increase in gene expression of proinflammatory cytokines (IL-1β, IL-6, and TNF-α), receptor-interacting serine/threonine-protein kinase 1 and 3 (RIPK1 and RIPK3) and the pseudokinase mixed-lineage kinase domain-like (MLKL). Immunofluorescence (IF) assays of cytochrome C oxidase I (COX1) confirmed mitochondrial dysfunction in aged cochleae, which correlated with the degree of mitochondrial morphological damage. IF assays also revealed localization and increased expression of RIPK3 in sensorineural tissues that underwent significant necroptosis (inner and outer hair cells and stria vascularis). Together, our data shows that the aging cochlea exhibits damaged mitochondria, enhanced synthesis of proinflammatory cytokines, and provides new evidence of necroptosis in the aging cochlea in in vivo.

## 1. Introduction

Approximately one in five people over the age of 50 has imperfect hearing, and almost half of those aged over 65 years have hearing difficulties [1,2,3,4]. Presbycusis, also termed age-related hearing loss (ARHL), is an irreversible hearing impairment associated with aging due to limited repair capacity of sensorineural tissues in the cochlea. Unfortunately, there is no effective cure for the patients, and future treatment development is still questionable due to lack of mechanistic insight [5]. In order to identify pathologic changes in the aged cochleae, we utilized C57BL/6J mice to investigate the pathophysiology of ARHL, as this strain displays accelerated, high-frequency hearing loss by 3–6 months of age and profound hearing impairment by 15 months of age [6,7,8,9,10,11].

Mitochondria are involved in the metabolic dysregulation associated with ARHL pathology. Morphologically, damage is apparent in the outer hair cells (OHCs) of animal ARHL models [10,12,13]. Mitochondria are the principal source of reactive oxygen species (ROS), the production of which is closely associated with ARHL progression. Antioxidants alleviate the deleterious effects of ROS and effectively treat oxidative stress-related diseases in an animal model of ARHL [14]. As mitochondria play key roles in both the respiratory chain and cell death, animal models of ARHL often exhibit defects in mitochondrial enzyme activities and mitochondrial-mediated apoptosis. Idh2-knockout mice exhibit accelerated ARHL progression, accompanied by a profound loss of hair cells and spiral ganglion neurons (SGNs), an increase in oxidative damage, and increased apoptotic cell death [15]. The mitochondrial proapoptotic BCL2-antagonist/killer 1 (Bak) gene mediates ARHL in C57BL/6J mice by enhancing mitochondrial fission and cellular apoptosis, both of which are systematic responses to oxidative stress [10]. 

Neuronal cell death occurs through various pathways in sensorineural tissue, leading to hearing impairment [2,5,10,16]. Necroptosis is a programmed cell death that exhibits necrosis-like morphological characteristics. Necroptosis is activated by the receptor-interacting protein (RIP) homology interaction motif (RHIM), and is mediated by proteins such as RIP3 and the mixed-lineage kinase domain-like (MLKL) protein [17]. Unlike apoptosis, necroptosis permeabilizes both intra- and extracellular membranes, releasing cellular and organelle contents into the extracellular medium and inducing inflammation. Necroptosis plays an important role in the pathogenesis of aging, neurodegenerative diseases, and hearing impairments including cisplatin- and aminoglycoside-induced ototoxicity and noise-induced hearing loss [17,18]. However, to the best of our knowledge, cochlear necroptosis has not been described in ARHL animal models. As successful modulation of cell death pathways may lead to potential development of clinically applicable drugs, we aimed to investigate the pathophysiology of ARHL by focusing on mitochondrial damage and necroptosis.

## 2. Materials and Methods

### 2.1. Experimental Animals and Design

All animal experiments were approved by Chungnam National University, Institutional Animal Care and Use Committee (IACUC, 9 January 2016). All animal care and use was conducted in accordance with the Guide for the Care and Use of Laboratory Animals. C57BL/6J male mice, aged 2 months or 20 months, were used in this study.

### 2.2. Auditory Brainstem Response (ABR)

ABR thresholds at frequencies between 4 and 32 kHz, and click sounds, were obtained separately from both ears as described previously [19]. The TDT System-3 (Tucker Davis Technologies, Gainesville, FL, USA) hardware and software were used to obtain the ABRs. The stimuli were computer-generated tone pips. The animals were anesthetized with intramuscular injection of zolazepam HCl 40 mg/kg (Zoletil, Virbac Animal Health, Carros, France) and xylazine 10 mg/kg (Rompun, Bayer Animal Health, Monheim, Germany). Subcutaneous needle electrodes were placed around the skull vertex and both infraauricular areas. Tone bursts, with a duration of 4 ms and rise-fall time of 1 ms at frequencies of 4, 8, 16, and 32 kHz, were used, in addition to clicks. The sound intensity was varied in 10 dB increments for the tone burst sounds and in 5 dB increments for the click and tone burst sounds close to the threshold. The contralateral ear was not masked because the stimuli were transmitted through a sealed earphone. The waveforms were analyzed using a custom program (BioSig RP, ver. 4.4.1; Tucker Davis Technologies) with the researcher blinded to the treatment group. Threshold was defined as the lowest stimulus intensity to evoke a wave III response > 0.2 μV.

### 2.3. Tissue Preparation and Immunofluorescence

Samples from mice aged 2 and 20 months were collected. Cochlear tissues were obtained to localize inner and outer hair cells and neuronal fibers as previously described [20,21]. Briefly, tissues were fixed in 4% paraformaldehyde in phosphate-buffered saline (PBS) for 1 h at room temperature to remove the cochlear bony walls and lateral wall tissues and separated individual cochlear turns. The remaining cochlear tissues were prepared within immunofluorescence. Tissues were blocked in 0.3% Triton X-100 (Sigma-Aldrich, St. Louis, MO) in 5% normal goat serum (Vector Laboratories, Burlingame, CA) for 1 h, then incubated with rabbit anti-myosin VIIa primary antibody (Proteus BioSciences, Ramona, CA)—Alexa Fluor 488 Phalloidin (A12379; Invitrogen-Molecular Probes, Eugene, OR); rabbit anti-CtBP2 primary antibody (BD Biosciences)—Alexa Fluor 594 Phalloidin (A11034; Invitrogen-Molecular Probes, Eugene, OR); chicken anti-NF-H primary antibody (Millipore)—Alexa Fluor 488 Phalloidin (A11039; Invitrogen-Molecular Probes, Eugene, OR); mouse anti-COX1 (also known as MTCO1) primary antibody (Invitrogen); rabbit anti-RIPK3 primary antibody (Novus Biologicals); or Hoechst33342 (H3570; Invitrogen-Molecular Probes, Eugene, OR) at a concentration of 1:200 in blocking solution overnight at 4 °C (1:1000 for Hoechst33342 for 1 min). After rinsing six times in PBS for 10 min, the tissues were incubated with each secondary antibody at a concentration of 1:200 in PBS for 2 h. The specimens were mounted on glass slides using Crystalmount (Biomeda, Foster City, CA) and observed under an epifluorescence microscope (Zeiss Axio Scope A1; Zeiss, Oberkochen, Germany) with a digital camera.

### 2.4. Transmission Electron Microscopy (TEM)

The decalcificated mouse cochlea were prefixed immediately in 2.5% glutaraldehyde–2% paraformaldehyde in 0.15 M sodium cacodylate buffer (pH 7.4) for 2 h at 4 °C. After washing with sodium cacodylate buffer, tissue samples were postfixed in 2% osmium tetroxide–1.5% ferrocyanide in 0.15M cacodylate buffer (pH 7.4) for 1 h. Then, samples were incubated with 1% TCH for 30 min and treated with 2% OsO4 for 30 min. Subsequently, samples were En bloc stained with 1% uranyl acetate overnight at 4 °C and lead citrate for 30 min at 60 °C. The tissues were then embedded in Epon 812 mixture after dehydration in an ethanol and propylene oxide series. Polymerization was conducted with pure resin at 70 °C for 24 h. Sections (200 nm) were obtained with an ultramicrotome (Ultra Cut-UCT, Leica, Vienna, Austria) and then collected on 100 mesh copper grids. The sections were visualized using conventional TEM (JEM-1400Plus) at 120 kV and Bio-HVEM (JEM-1000BEF, JEOL, Tokyo, Japan) at 1000 kV. The sections were visualized using Bio-HVEM system (JEM-1400Plus at 120 kV and JEM-1000BEF at 1000 kV, JEOL, JAPAN).

### 2.5. Measurement of Cochlear Blood Flow

The left tympanic bulla of each mouse was exposed and opened under anesthesia. After the mouse was placed on the stereotaxic instrument, the cochlear blood flow was measured using a 0.1 mm diameter laser Doppler probe placed over the lateral wall of the cochlea. Cochlear blood flow was determined from an intensity oscillation that was translated from the frequency of the oscillation produced by the Doppler frequency shift of the red blood cells in the left tympanic bulla, using a Laser Doppler Flowmeter (Transonic Systems, Ithaca, NY, USA). Each intensity oscillation was measured separately, and relative cochlear blood flow was reported as the ratio of the control (pre) value to the postnoise exposure value.

### 2.6. Quantitative Real Time Polymerase Chain Reaction (qRT-PCR) 

Quantitative RT-PCR was performed as previously described [19,22]. Briefly, tissues were collected and frozen immediately in liquid nitrogen and homogenized. Total RNA was extracted with TRIzol reagent (Thermo Fisher Scientific, Waltham, MA USA) according to the manufacturer’s protocol. RNA was quantified using a Nano drop (Nano Drop Technologies, Wilmington, DE). cDNA was produced using the cDNA synthesis kit (Roche, Branchburg, NJ, USA). Real time PCR was performed on a CFX Connect Real-Time PCR Detection System (BioRad, Des Plaines, IL, USA) by using a reaction mixture with SYBR Green as the fluorescent dye (Applied Biosystems, Waltham, Massachusetts, USA), a 1/10 vol of the cDNA preparation as template, and 250 nM of each primer (Realtime primers, PA). The fold change in the target gene relative to endogenous control gene (glyceraldehyde 3-phosphate dehydrogenase, GAPDH) was determined by: fold changeXX = 2^−Δ(ΔC^_T_^)^ where ΔC_T_ = C_T,target gene_ − C_T,GAPDH_ and Δ(ΔC_T_) = ΔC_T,Aged cochlea_ − ΔC_T,Young cochlea_ [23].

### 2.7. Image Processing and Statistical Analysis

Adobe Photoshop CS6 was used for adjustment of image contrast, superimposition of images, and colorization of monochrome fluorescence images. An unpaired Student’s *t*-test was used for all comparisons. A *p*-value < 0.05 was significant in each case. All tests were performed using GraphPad Prism 6.

## 3. Results

### 3.1. Auditory Brainstem Response Thresholds and Histopathologies of Young and Aging Cochleae

**Figure 1:**
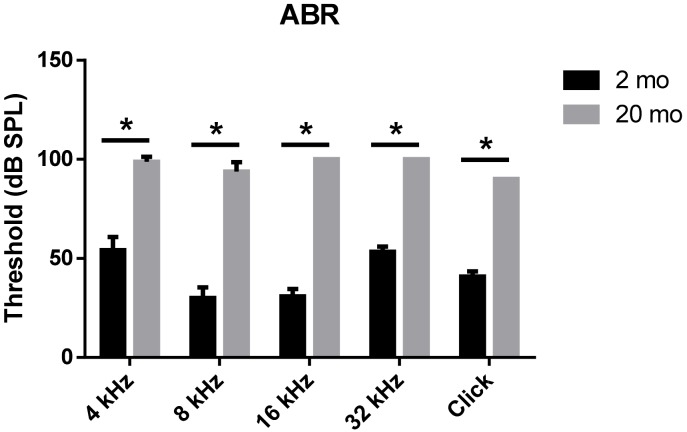
Aged mice displayed greater hearing loss as compared to young animals. ABR Table; 2-month- or 20-month-old male C57BL/6J mice. Aged mice exerted a significantly increased ABR threshold compared to young mice at all frequencies and click sound. The graph represents mean ± S.E.M. *t*-test. * *p* < 0.0001. *n* = 6, 2 months; *n* = 4, 20 months.

**Figure 2:**
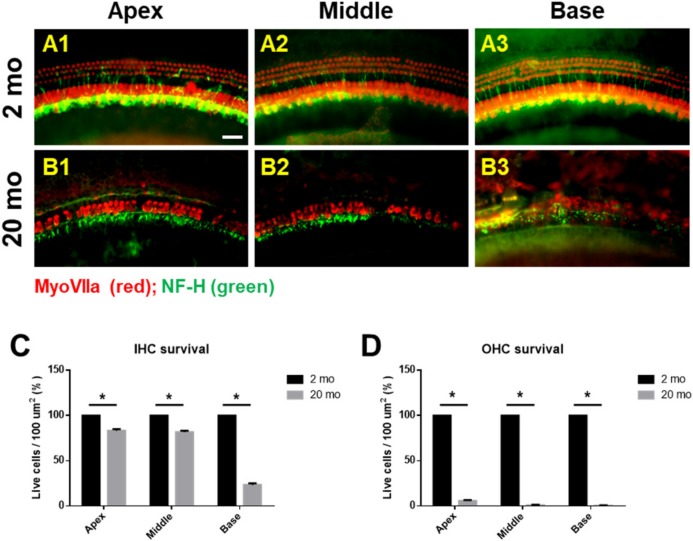
Cochlear hair cells are significantly lost in aged mice. Whole-mount preparations of the auditory epithelium in the 2 month-(**A1**–**A3**) and 20 month-old (**B1**–**B3**) mice. Tissues were stained for myosin VIIa (red) to visualize the hair cells and then photographed using epifluorescence. (**A**,**B**) OHCs were more noticeably destroyed on the middle and basal turns of the cochlea in the aged mice (B1–B3) compared to the young mice (**A1**–**A3**). (**C**,**D**) Quantitative analysis of hair cell survival on IHCs (**C**) and OHCs (**D**): apex, middle, and basal turns. A1 and B1, apical turn; A2 and B2, middle turn; A3 and B3, basal turn; OHC, outer hair cell; IHC, inner hair cell. Scale bar = 30 μm. All graphs represent mean ± S.E.M. * *p* < 0.05. Unpaired *t*-test. *n* = 3.

We first evaluated age-related functional impairment of hearing in male C57BL/6J mice aged 2 or 20 months. As shown in Figure 1, 20-month-old mice (*n* = 4) exhibited significantly increased auditory brainstem response (ABR) thresholds at 4, 8, 16, and 32 kHz and when click sounds were delivered (*t*-test, **** *p* < 0.0001). Whole-mount analyses of the auditory epithelium (Figure 2) confirmed that both inner hair cells (IHCs) (Figure 2A1–A3) and outer hair cells (OHCs) (Figure 2B1–B3) were more damaged in aging mice than in young mice. However, damage to the OHCs of all cochlear turns was more prominent than the damage to IHCs. Quantitative analyses of IHC (Figure 2C) and OHC (Figure 2D) survival indicated extensive loss of both cell types in aging mice; OHC loss was more marked than IHC loss (unpaired *t*-test, *n* = 3, * *p* < 0.05).

**Figure 3:**
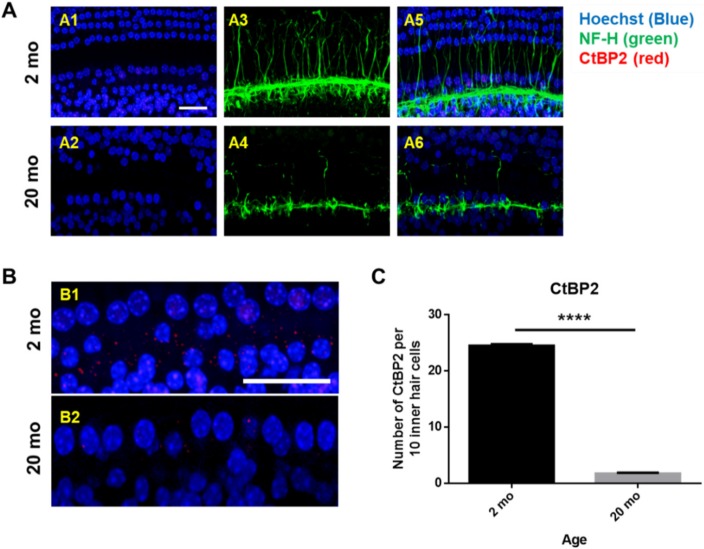
Severe synaptopathy and loss of nerve fibers were observed in aged mice. Whole-mounts of auditory epithelium were triple-stained with Hoechst (blue, nuclear marker, **A1** and **A2**), NF-H (green, neuronal cell marker, **A3** and **A4**) and CtBP2 (red, a marker of synaptic ribbons, A5 and A6) to evaluate synaptopathy (**A5** and **A6**, merged). (**A1,A3**,**A5**) Hair cells (blue) and neurons (green) remain intact in young mice. (**A2**,**A4**,**A6**) Neuronal filament in auditory epithelium (green, A4) are notably destroyed in the aged mice. (**B**) Many of the IHC and OHC (blue, Hoechst) are significantly diminished in aged animals compared to young mice. (**C**) Number of presynaptic marker (CtBP2, red) per 10 hair cells was quantified. Aging mice had a significant decrease in CtBP2 counts as compared to the young, indicating significant loss of a marker of synaptic ribbons in aging animals. Scale bar = 30 μm. Graphs represent mean ± S.E.M. **** *p* < 0.0001. Unpaired *t*-test. *n* = 3.

Next, whole mounts of the auditory epithelium were triple-stained with a Hoechst stain specific for nuclei, fluorescence-tagged antibodies against neurofilament-H (NF-H), and C-terminal binding protein 2 (CtBP2; a marker of synaptic ribbons), to evaluate synaptopathy. Figure 3A is a representative photograph. Hair cells (blue) and neuronal filaments (green) remained intact in young mice, consistent with the results shown in Figure 2, but the numbers of IHCs and OHCs (stained blue by Hoechst) were significantly reduced in aging mice. Neuronal filaments of the auditory epithelium (green, Figure 3A) were notably damaged in aging cochlea. The numbers of presynaptic marker counts (Figure 3B,C; CtBP2/10 inner hair cells) were significantly lower in aging mice than in young mice (unpaired *t*-test, ****p < 0.0001, *n* = 3), indicating severe synaptopathy and nerve fiber loss in aging cochleae. 

### 3.2. Cochlear Microcirculation and Morphologies

**Figure 4:**
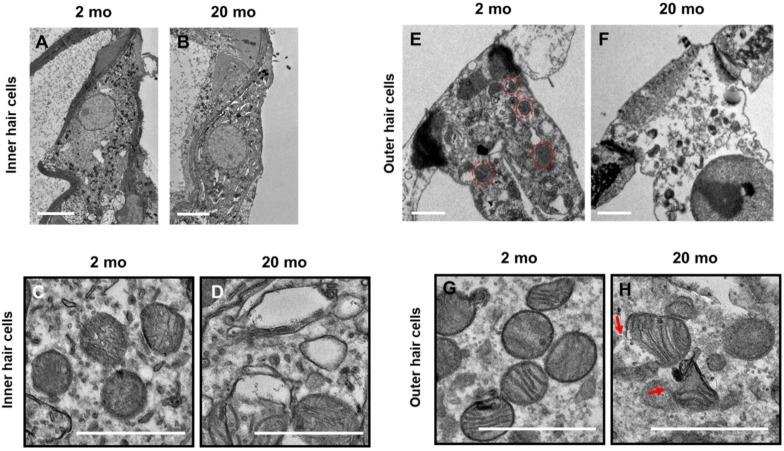
Ultrastructural analysis of cochlear inner and outer hair cells from young and aged mice. (**A**–**D**) Young inner hair cells show normal appearing mitochondria (**A**) with well-defined lamellar cristae (**C**), while old ones present numerous vacuoles in the cytoplasm (**B**) and damaged mitochondria with absent or disorganized cristae (**D**). (**E**,**G**) Normal outer hair cells with distinct mitochondrial cristae from young cochlea. (**F**) Outer hair cells of aging cochlea show “swollen” cell body and chromatin compaction. Their mitochondria appear enlarged and present altered cristae and a broken/bursting wall (**H**), arrows). Scale bar of (**A**,**B**) represents 5 μm and of C–H represents 1 μm.

**Figure 5:**
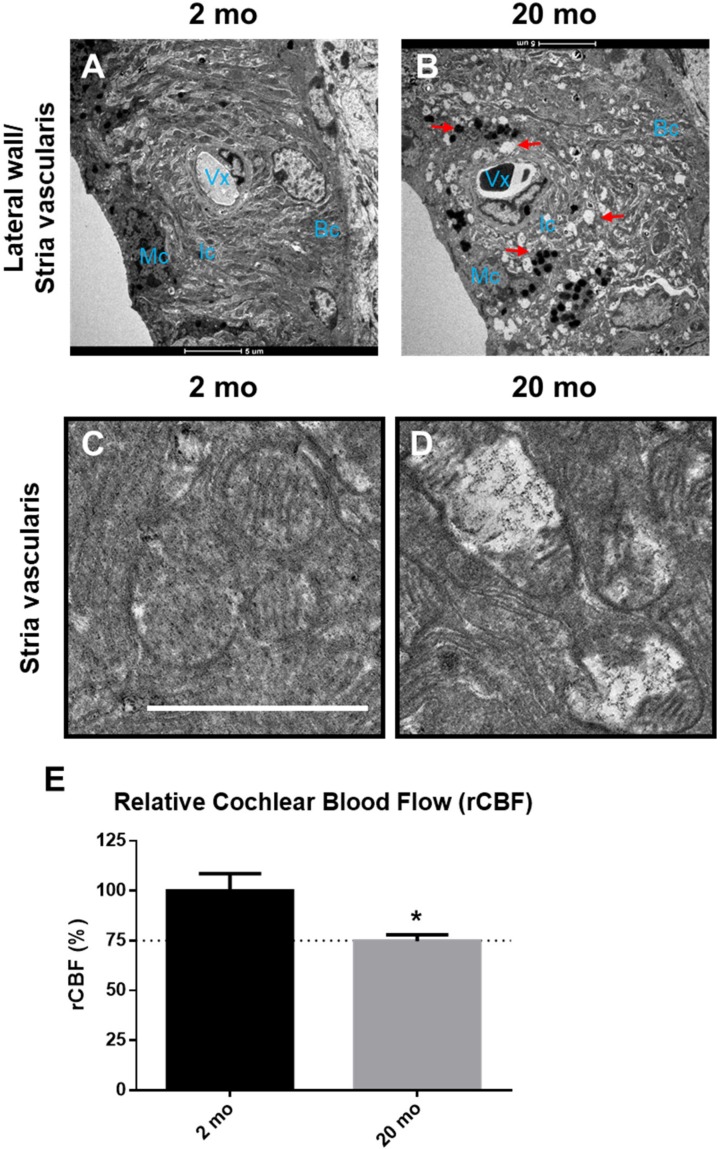
Microscopic images of lateral wall and cochlear blood flow of young and aging animals. (**A**) Young stria vascularis images taken by transmission electron microscopy (TEM) shows the three layers of strial cells, marginal (Mc), intermediate (Ic), and basal (Bc) cells, and the blood vessels (Vx) appear normal. (**B**) The stria vascularis degeneration in aging cochlea, as indicated by numerous vacuoles (red arrows), pigmentation (red arrows) and enlarged intercellular spaces, is obvious. Scale bar of A–B represents 5 μm. (**C**) Normal mitochondria with distinct cristae in young stria vascularis. (**D**) Damaged mitochondria with degenerated cristae in aging stria vascularis. Scale bar of C,D represents 1 μm. (**E**) Cochlear blood flow was measured using a 0.1 mm diameter laser Doppler probe placed over the lateral wall of the cochlea of the 2 and 20 months old C57BL/6J male mice. Significantly decreased blood flow were observed in aging cochlea. Graphs represent mean ± S.E.M. * *p* < 0.05. Unpaired *t*-test. *n* = 5–8. Graphs represent mean ± S.E.M. * *p* < 0.05. *t*-test. *n* = 4.

Transmission electron microscopy (TEM) was conducted to investigate the morphological changes of the hair cells (Figure 4) and stria vascularis (Figure 5) in young (2 months) and aged (20 months) mice. Young inner and outer hair cells (2 months) showed a healthy appearance and normal mitochondrial morphology with well-defined cristae (Figure 4C,G). At 20 months of age, both IHCs (Figure 4B) and OHCs (Figure 4F) had numerous vacuoles in the cytoplasm. Aging mitochondria exhibited irregular sizes and electrolucent central matrices with undistinguishable, absent, and/or nonparallel disorganized cristae (Figure 4D,H). Ultrastructural analysis of cochlear lateral wall architecture revealed stria vascularis degeneration in aging cochleae, as indicated by numerous vacuoles and enlarged intercellular spaces (red arrows, Figure 5B) in all three layers of strial cells, marginal (Mc), intermediate (Ic), and basal (Bc) cells. Compared to young mice, aged stria vascularis exhibited damaged mitochondria with disorganized dysmorphic cristae (Figure 5D). To explore how aging changed cochlear microcirculation, blood flow was measured using laser Doppler flowmetry. Cochlear blood flow was significantly lower in aging mice than in young mice (Figure 5E, * *p* < 0.05, unpaired *t*-test). Thus, mitochondrial morphological changes in organ of Corti and lateral wall may contribute to the hearing loss and blood flow reduction found in ARHL.

### 3.3. Mitochondrial Damage and Stress Responses

**Figure 6:**
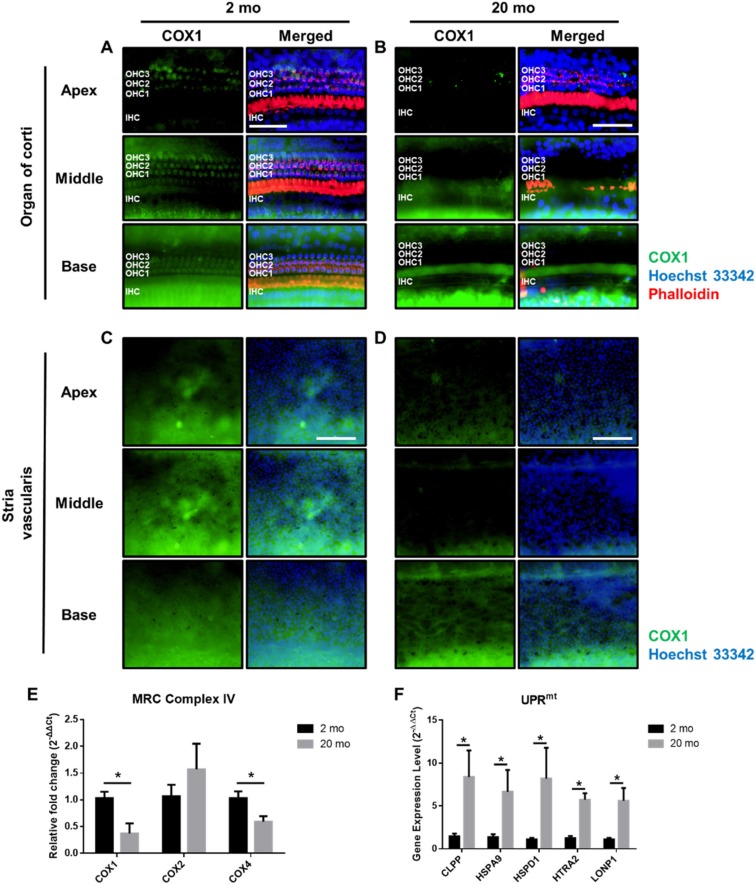
Mitochondrial respiratory chain complex IV and stress responses in young and aged mice. (**A**–**D**) Whole mounts of the auditory epithelium were triple-stained with a Hoechst33342 (blue) specific for nuclei, fluorescence-tagged antibodies against cytochrome C oxidase I (COX1, green) and phalloidin (red) from apex, middle, and base turns of organ of Corti and stria vascularis. Aged auditory epithelium (**A**,**B**) and stria vascularis (**C**,**D**) show a decreased COX1 protein expression. Scale bar represents 100 μm. (**E**) Genes in mitochondrial respiratory chain complex IV in young and aged mice. Aging cochlea expresses significantly decreased COX I and COX IV levels. (**F**) Gene expression levels of mitochondrial unfolded protein response (UPR) markers in young and aged cochlea. GAPDH was used as a reference gene. Graphs represent mean ± S.E.M. * *p* < 0.05. Unpaired *t*-test. *n* = 5–8. IHC, inner hair cell; OHC1-3, three rows of outer hair cells.

Cochlear mitochondrial damage/dysfunction is a principal cause of sensorineural hearing deficits, including drug-induced or age-related hearing loss [16,24,25,26,27]. We next investigated whether mitochondrial function in aging cochlea is also disturbed and correlates with the degree of mitochondrial damage. To measure the overall cochlear mitochondrial contents, we used qRT-PCR to amplify mRNAs encoding genes of the mitochondrial respiratory chain complexes I–V in cochleae (Figure 6A). Cytochrome c oxidase (COX) of complex IV plays an essential role in energy production [28]. COX4 is a nuclear-encoded subunit of cytochrome oxidase c, the terminal enzyme of the electron transport chain located on the inner mitochondrial membrane [28,29]. Aging cochleae exhibited lower levels of cytochrome c oxidase subunits 1 (COX1, *p* = 0.0171) and 4 (COX4, *p* = 0.0357), indicating that the mitochondria of the aging cochleae were dysfunctional. Note that we did not see any significant changes between young and aging cochleae in complexes I, II, III, and V (Figure S1A–D).

We next utilized IF assays of COX1 to confirm both the localization and protein expression in the subregions of cochleae. Whole mounts of the auditory epithelium were triple-stained with Hoechst33342 specific for nuclei (blue), fluorescence-tagged antibodies against cytochrome C oxidase I (COX1, green) and phalloidin (red) (Figure 6A–D). Cells stained blue by Hoechst and phalloidin remained intact in young mice in all three turns (apex, middle and base) of the organ of Corti (Figure 6A) and stria vascularis (Figure 6C), consistent with the previous results (Figure 2 and Figure 3). We also found that COX1 expression was prominent in these cells (left panel of Figure 6A,C). However, the numbers of cells stained by Hoechst and phalloidin were significantly reduced in aged mice (right panel of Figure 6B,D) and COX1 protein expression was significantly reduced in all turns of the auditory epithelium and stria vascularis. Notably, COX1 expression completely disappeared from sensory hair cells in the middle turn of organ of Corti (middle panel of Figure 6B, COX1 negative hair cells).

The mitochondrial network not only serves as the signaling core for the oxidative response and apoptotic pathways, but is also required for the production of energy and essential cofactors. Multiple mechanisms have been developed to maintain the health of the organism. One such pathway is the mitochondrial unfolded protein response (UPR^mt^), which removes misfolded proteins to restore protein homeostasis. To examine how the aging cochlea coped with mitochondrial stress, we quantified marker genes of the UPR^mt^ (Figure 6). The aging cochlea exhibited significantly increased levels of UPR^mt^-related genes, including *CLPP*, which encodes the caseinolytic mitochondrial matrix peptidase proteolytic subunit, *HSPA9*, which encodes the heat shock 70 kDa protein; *HSPD1*, which encodes the heat shock 60 kDa protein 1; *HTRA2*, which encodes htrA serine peptidase 2; and *LPNP1*, which encodes mitochondrial ion peptidase 1. These data indicate that aging cochleae exhibited a reduced overall level of mitochondria, many of which showed morphological damage, consistent with the hypothesis that energy production was compromised.

### 3.4. Aging Cochleae Exhibit Necroptosis and An Inflammatory Response

**Figure 7:**
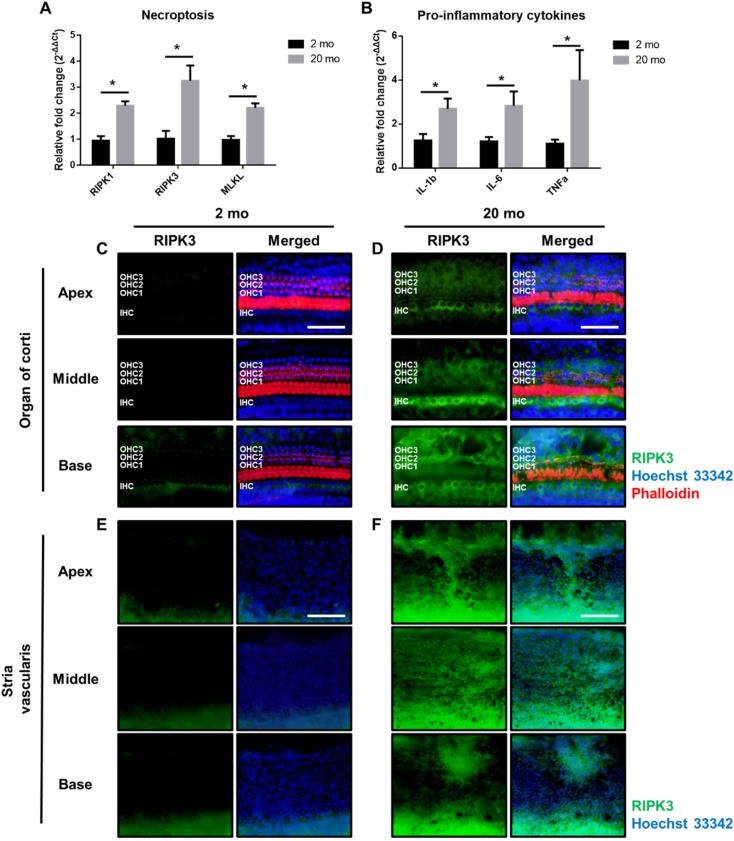
Aging cochlea exerts necroptosis and inflammatory responses. (**A**–**B**) Gene expression levels of necroptosis (**A**) and proinflammatory cytokines (**B**) and were quantified by qRT-PCR. Graphs represent mean ± S.E.M. * *p* < 0.05. Unpaired *t*-test. *n* = 6–8. (**C**–**F**) Whole mounts were triple-stained with a Hoechst33342 (blue) specific for nuclei, fluorescence-tagged antibodies against RIPK3 (green) and phalloidin (red) from apex, middle, and base turns of organ of Corti and stria vascularis. Aged cochlea and stria vascularis present significantly increased RIPK3 induction as compared to young ones. Scale bar represents 100 μm. IHC, inner hair cell; OHC1-3, three rows of outer hair cells.

Next, we investigated how sensorineural tissues undergo necroptosis, a regulated form of cell death. Unlike other cell death mechanisms including apoptosis, autophagy, and necrosis, necroptotic cell death is not well-defined in ARHL. Necroptosis is a form of programmed cell death that exhibits necrosis-like morphological features. Necroptosis is associated with the permeabilization of cellular membranes, the release of cellular and organelle contents into the extracellular medium, and inflammation [17,30,31,32]. The most defined molecular pathway of necroptosis is mediated by TNF-α receptor through receptor-interacting serine/threonine-protein kinase 1 and 3 (RIPK1 and RIPK3) and the pseudokinase mixed-lineage kinase domain-like (MLKL). We subjected whole cochlear homogenates to qRT-PCR to measure the levels of necroptosis and the inflammatory response. The aging cochleae exhibited significantly increased levels of all necroptosis marker genes, including *RIPK1*, *RIPK3*, and *MLKL* (Figure 7A), and increased levels of the proinflammatory cytokines IL-1β, IL-6, and TNF-α (Figure 7B).

The observed decrease of RIPK3 expression was confirmed with immunofluorescence analysis. Whole mounts of the auditory epithelium were triple-stained with a Hoechst33342 specific for nuclei (blue), fluorescence-tagged antibodies against RIPK3 (RIPK3, green), and phalloidin (red) (Figure 7C–F). Sensory cells, stained by Hoechst33342 and phalloidin, appeared intact in young mice in all three turns (apex, middle, and base) of the organ of Corti (Figure 7C) and stria vascularis (Figure 7E), consistent with the results shown in Figure 2, Figure 3 and Figure 6. RIPK3 was absent or minimally expressed (left panel of Figure 7C,E). In contrast, RIPK3 protein expression was prominent in the surviving sensory and strial cells in old aged mice (left panel of Figure 7D,F). Our results suggest that the aging cochleae exhibit significant proinflammatory and necroptotic responses when compared to the young cochleae, thus providing novel evidence that necroptosis is involved in cochlear aging in vivo.

## 4. Discussion

We identified increased RIPK3 level in the aging cochlea, especially in the inner and outer hair cells and stria vascularis. Pronounced reduction in COX1 correlated with the degree of mitochondrial morphological damage and hearing impairment found in aging animals was associated with a loss of sensory hair cells and neuronal filaments. Our data suggest that mitochondrial degeneration and necroptosis may play a critical role in the pathophysiology of ARHL and provide mechanistic insights for future therapeutic development.

Laboratory animals are useful when investigating ARHL because of their short lifespans and well-defined genetics. Like humans, many inbred mouse strains show variable extents of ARHL; the age of onset ranges from 3 months in DBA/2J mice to over 20 months in CBA/CaJ mice [27,33,34,35]. The C57BL/6J and CBA/CaJ strains are the inbred strains most widely used in hearing research [33]. The C57BL/6J strain has been extensively employed as a model of early-onset ARHL; the mice exhibit high-frequency hearing loss by 3–6 months and profound hearing impairment by 15 months [6,7,8,9]. In contrast, CBA/CaJ mice exhibit normal hearing to 15 months or more, and are often used as positive controls [6,34,36]. One well-documented genetic factor responsible for hearing loss in C57BL/6J mice is the recessive *ahl* allele of *Cdh23*, which encodes cadherin 23 [37]. However, interestingly, inbred strain variants of *Cdh23* show differences in ARHL onset and progression: the CBA/CaJ-derived *Cdh23^Ahl+^* allele dramatically reduces hair cell death and hearing loss in a C57BL/6J genetic background, but the C57BL/6J-derived *Cdh23^ahl^* allele has little effect on hearing loss in a CBA/CaJ background [33]. *Cdh23^ahl^* homozygosity is necessary but not sufficient to trigger accelerated hearing loss in C57BL/6J mice [9,33]. An interesting study by Frisina et al. showed that F1 (CBA × C57) hybrids exhibit better hearing (“golden ears”) than either parental strain [38]. Despite the accelerated hearing loss of the C57BL/6J strain, such mice are valuable when studying the features of presbycusis; we used these mice in our current and earlier studies [10,11,39,40,41,42,43,44,45,46,47,48]. The early-onset hearing loss of C57BL/6J mice endures for the lifespan, fitting well with the reality of human hearing loss. The World Health Organization estimated that ~466 million people worldwide, including 34 million children, have some degree of hearing loss.

Necroptosis triggers inflammation and cell death caused by cell lysis. Increasing evidence suggests that necroptosis plays a critical role in the pathogenesis of several neurodegenerative diseases and manifestations of hearing impairment, including cisplatin- and aminoglycoside-induced ototoxicity and noise-induced hearing loss [17,18,49,50,51]. Inhibition of necroptosis has been reported to confer neuroprotective effects in animal models of neurodegenerative disorders, and necroptotic factors may thus be promising therapeutic targets [17]. We provide the first evidence that the aging cochlea exhibits necroptosis in vivo. Few other studies have investigated the role of necroptosis in the cochlea; in these studies, models of ototoxicity and noise-induced hearing loss were employed, but not a model of age-related hearing impairment. Necrostatin-1 (Nec-1, an RIPK1 inhibitor) alleviated noise-induced hearing loss in the mouse [52], protected spiral ganglion neurons, and improved ABR thresholds in rats exposed to ouabain [53]. Park et al. reported that NecroX, a necroptosis inhibitor, prevented gentamicin-induced HC loss in neonatal mouse explants of the organ of Corti [54]. Ruhl et al. reported that in vivo, both necroptosis and apoptosis are involved in cisplatin- and aminoglycoside-induced ototoxicity in both sexes, but, ex vivo, only apoptosis contributed to the ototoxicity [51] evident in Casp8 and Ripk3 knockout models. The authors thus confirmed earlier genetic evidence that Caspase-8-mediated (extrinsic) apoptosis is involved in cisplatin-mediated ototoxicity. We also show that aging cochleae undergoes at least two programmed cell death pathways simultaneously, apoptosis (Figure S1F–G) and necroptosis. However, the relative contributions of these pathways to ARHL and cochlea aging are still unknown.

Inflammaging (chronic low-grade inflammation) is a hallmark of aging and is a major risk factor for a variety of age-related diseases, including neurodegenerative diseases, cardiovascular disease, and type 2 diabetes. Despite the strong association among inflammation, aging, and age-associated diseases, the molecular mechanisms that contribute to the chronic, low-grade inflammation observed in aging animals remain unknown. Our initial aim was to explore whether aging affects the sensory, neural, and metabolic features of the cochlea through age-related inflammation and necroptotic stress. We found an association between inflammation and necroptosis in the aging cochlea. Significantly increased necroptosis protein, RIPK3, was significantly increased in the organ of Corti and stria vascularis. Recent studies have identified both resident macrophages (CD163-, IBA1-, and CD68-positive cells) and migrated macrophages in the human cochlea [55,56]. It is well-known that IL-1 and IL-6 are important modulators of the innate and adaptive immune responses [57]. Thus, it would be interesting to explore whether resident or infiltrated immune cells are responsible for the increased cytokine levels, and, where the resident macrophages, if present, are located within the cochlea (i.e.; in the lateral wall, auditory nerve, or elsewhere).

The proinflammatory cytokine TNF-α and its receptor, TNFR (TNF-α receptor), play key roles in cell death machineries, including necroptosis and apoptosis [58]. Chen et al. reported that TNFα-induced necroptosis in murine fibrosarcoma L929 cells resulted in enriched levels of RIP1/RIP3/MLKL in the mitochondrial associated membrane fraction of cells [59]. Others show that knockdown or inhibition of *Drp1* (dynamin-related protein 1; also known as *DNM1L*, dynamin-1-like protein) protects both HeLa and HT-29 cells from TNFα-mediated necroptosis [60]. Notably, we also observed a significant increase of *Drp1* expression in aging cochleae in our in vivo system (Figure S1E).

Our study also contains several weaknesses. First, protein expression levels were measured only by IF assays, and not by Western blot (WB) or Elisa. We employed the IF assays to investigate protein expression in subregions of cochlea rather than detecting signals from the whole cochlea. Another weakness is lack of mitochondrial function studies (e.g.; mitochondrial enzymatic activity) to confirm the mitochondrial dysfunction in ARHL. Finally, although we present two main phenomena (mitochondrial damage and necroptosis) in the aging cochlea, we are unable to suggest a direct relation between mitochondria/ROS and necroptosis. Despite the long assumption that ROS and mitochondria are involved with necroptosis, a previous study showed that necroptosis can occur in the absence of mitochondria or ROS [61]. Further studies are necessary to examine which specific genes and pathways are involved with the necroptotic cell death in ARHL models; and whether the increase in chronic inflammation actually causes cochlear aging and age-related hearing loss.

## Supplementary Materials

The following are available online at https://www.mdpi.com/1422-0067/21/7/2505/s1.

## Abbreviations

ABR	Auditory brainstem response
ARHL	Age-related hearing loss
Bak	BCL2-antagonist/killer 1
Bc	Basal cells
Cdh23	Cadeherin 23
COX	Cytochrome c oxidase
COX1	Cytochrome C oxidase I
COX4	Cytochrome C oxidase Ⅳ
CLPP	Caseinolytic mitochondrial matrix peptidase proteolytic subunit
DNM1L	Dynamin-1-like protein
Drp1	Dynamin-related protein 1
GAPDH	Glyceraldehyde-3-phosphate dehydrogenase
HSPA9	Heat shock 70-kDa protein
HSPD1	Heat shock 60-kDa protein 1
HTRA2	HtrA serine peptidase 2
Ic	Intermediate cells
IHC	Inner hair cells
LPNP1	Mitochondrial ion peptidase 1
Mc	Marginal cells
MLKL	Mixed-lineage kinase domain-like
Nec1	Necrostatin-1
OHCs	Outer hair cells
PBS	Phosphate-buffered saline
qRT-PCR	Quantitative real time polymerase chain reaction
RHIM	RIP homology interaction motif
RIP	Receptor-interacting protein
ROS	Reactive oxygen species
SGNs	Spiral ganglion neurons
TEM	Transmission Electron Microscopy
UPR	Unfolded protein response
UPR^mt^	Mitochondrial unfolded protein response
