# Supplementary material for: Mitochondrial Damage and Necroptosis in Aging Cochlea
Source: Int J Mol Sci. 2020 Apr 3;21(7):2505. doi: 10.3390/ijms21072505 (PMC7177801; doi:10.3390/ijms21072505)
Supplement: Supplementary file 1 [file ijms-21-02505-s001.pdf]

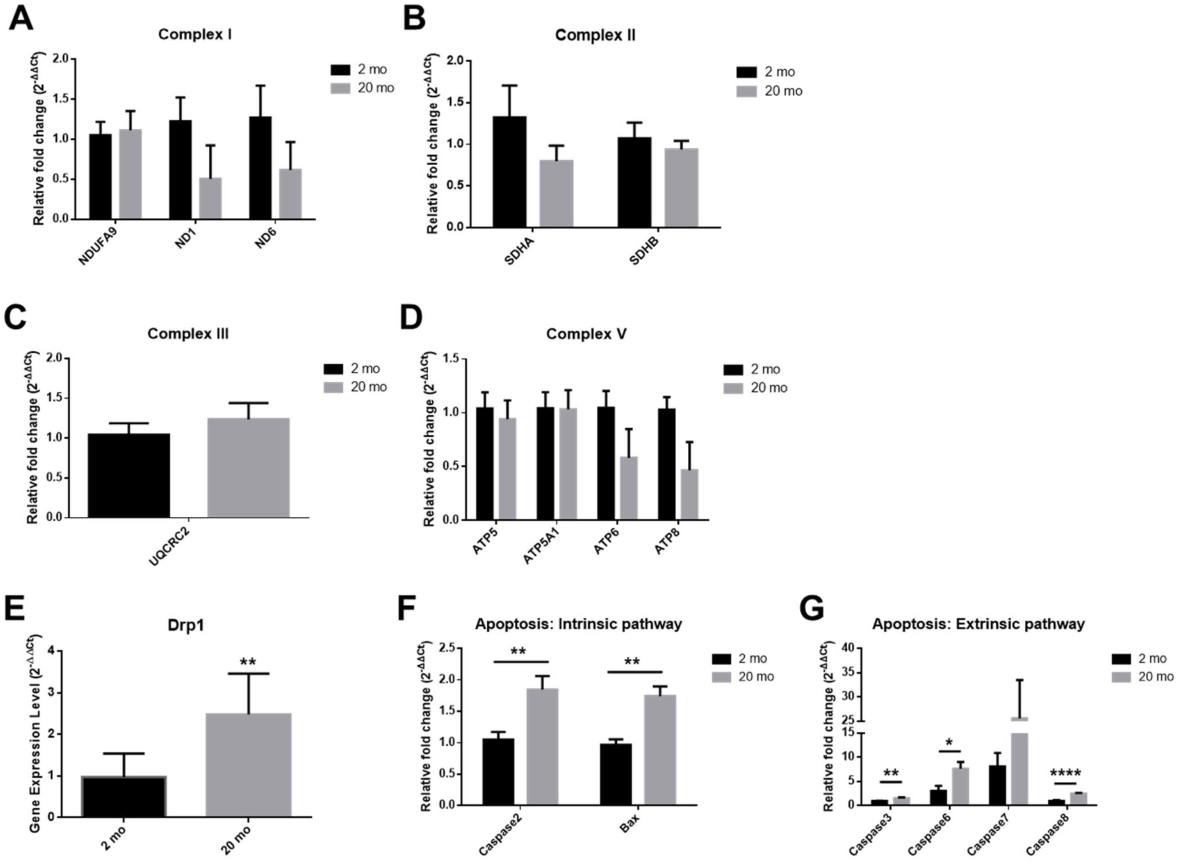

Supplementary Figure 1. Mitochondrial respiratory chain complexes-, dynamin-related protein 1 (Drp1)-, and apoptosis-related genes in young and aging cochleae. qRT-PCR for genes related to mitochondrial respiratory chain complexes (**A**–**D**), *Drp1* (**E**) and intrinsic (**F**) and extrinsic (**G**) pathways of apoptosis in young (2 months old) and aged (20 months old) mice. Graphs represent mean ± S.E.M. * $p < 0.05$, ** $p < 0.01$, **** $p < 0.0001$. Unpaired *t*-test. $n = 5–8$.
